# Rare Causes of Hydronephrosis in Adults and Diagnosis Algorithm: Analysis of 100 Cases During 15 Years

**DOI:** 10.7759/cureus.8226

**Published:** 2020-05-21

**Authors:** Musab Ilgi, Göksel Bayar, Elsad Abdullayev, Sedat Cakmak, Hüseyin Acinikli, Sinan L Kirecci, Kaya Horasanli

**Affiliations:** 1 Urology, Hopa State Hospital, Artvin, TUR; 2 Urology, Mardin State Hospital, Mardin, TUR; 3 Urology, Private Atlas Hospital, Istanbul, TUR; 4 Urology, Haseki Training and Research Hospital, University of Health Sciences, Istanbul, TUR; 5 Urology, Mersin District Hospital, Mersin, TUR; 6 Urology, Sisli Hamidiye Etfal Training and Research Hospital, University of Health Sciences, Istanbul, TUR; 7 Urology, Sisli Hamidiye Etfal Research and Training Hopsital, University of Health Sciences, Istanbul, TUR

**Keywords:** hydronephrosis, urinary system obstruction, nephropathy

## Abstract

Introduction

Hydronephrosis (HN) is a common pathology that is with/without obstruction. HN should be promptly addressed; otherwise, it progresses to cause impaired kidney function. This study was conducted to define the diagnosis algorithm and poor prognostic parameters used to evaluate unknown HN.

Materials and Methods

This study enrolled 100 patients who were over 20 years of age and were admitted to the center between 2001 and 2015 for the diagnosis and treatment of HN. Although initial diagnostic tests were applied, the HN etiology of the patients could not be found in ambulatory conditions; therefore, they were hospitalized to seek the causes for their HN. Patients who had a malignancy or tuberculosis or any previous ureteral injury were excluded.

Results

Of these cases, 29 were on both sides, whereas 42 were on the left side. Despite further investigations, the etiology was not determined in five patients. The frequency of malignancy in patients with hematuria (5/15) was two times higher than in patients without hematuria (33% vs. 17.6%; p = 0.01). Additionally, the malignancy rate was significantly higher in patients with weight loss (100%) than those without weight loss (OR: 6.25; p < 0.001).

Conclusions

Further investigation is recommended to define the precise etiology of HN. Hematuria and weight loss should be considered poor predictive factors during diagnosis.

## Introduction

Hydronephrosis (HN) is the dilatation of the renal pelvis and/or calyces. Dilatation in the ureter is called hydroureteronephrosis (HUN). HN is classified according to the level of and the side of the obstruction [1], but the cause of HN may also be with/without obstruction. The functional or anatomical obstruction causes the urine to back up into the kidney, causing accumulation, pelvicalyceal distention, and nephropathy. HN should be addressed quickly; otherwise, HN can lead to severely impaired kidney function and kidney failure.

The incidence of HN in an autopsy series was found to be 3.1%. Its prevalence is similar in men and women until 20 years of age, but it is more common in women between 20 and 60 years of age, possibly due to pregnancy and gynecological malignancy. After 60 years of age, it is more common in males, probably due to prostatic diseases [2]. However, developing obstructions are cured with treatment; therefore, the actual incidence of HN is not known, and a study has yet to be published in the literature.

The first imaging modalities to be performed in suspected dilatation are ultrasonography (US) and computed tomography (CT). However, no precise algorithm for diagnosis exists. Instead, the approach is based on the patient clinic and the type of HN [1]. Therefore, this study aimed to establish a diagnosis algorithm and detect the poor prognostic factors in patients diagnosed with unknown causes of HN in ambulatory conditions.

## Materials and methods

Patients who were over 20 years of age and were admitted to the authors’ center between 2001 and 2015 for the diagnosis and treatment of HN were retrospectively reviewed. An exact etiology could not be found for these patients in outpatient clinics; therefore, they were hospitalized to investigate the causes behind their HN.

The first presenting symptoms were urological flank pain, lower urinary tract symptoms (LUTSs), blood in the urine, or non-specific troubles such as abdominal pain, palpitations, fever, fatigue, nausea, and weight loss. Weight loss is considered an involuntary loss of 10% of current weight in the last six months or 5% loss in the last month. CT and US examinations were performed on all patients on the first visit. CT urography (CTU) was used for those who could not be diagnosed and did not have any contraindications to the use of contrast agents. Cystoscopy was performed on all patients who had complete HUN. Prostate-specific antigen (PSA) levels were tested in male patients, and prostate biopsy was performed in male patients when indicated. After these procedures, the patients who could not be diagnosed were admitted to the hospital.

The first 100 patients who met all these criteria, whose data were complete, and who were at least three months follow-up and recently hospitalized were enrolled in this study. Patients who were diagnosed with malignancy or tuberculosis or had any previously known ureteral injury were excluded from the survey. The relation with the etiology of HN was examined regarding the mentioned factors.

HN was classified as bilateral or unilateral, concomitant ureteral dilatation or isolated, incomplete or complete HUN, and due to internal or external causes. The patients were examined, and findings were surveyed to find the rare causes of HN in urology practice. A Pearson chi-square test was used for statistical analysis; p < 0.05 was accepted as a significant value.

## Results

The median age of the patients was 49 years (range: 22-75 years), and 58 were female. Of the HN cases, 29 were bilateral, whereas 42 of the 71 unilateral cases were on the left side. Despite further investigations, the cause of HN could not be determined in five patients; these cases were identified as non-reflux non-obstructive dilated ureter. HN was observed in the upper urinary system in 50 cases, in the lower urinary system in 11, and due to non-urinary causes in 23 patients. Additionally, 10 patients with urinary tuberculosis and one with urinary candidiasis were classified (Table 1).

**Table 1:** Patients’ details

No. of patients	100
Median age (minimum-maximum)	49 (22-75)
Gender (female/male)	58/42
Laterality (unilateral/bilateral)	71/29
Side on unilateral (left/right)	42/29
Hydronephrosis (incomplete/complete)	12 (49/39)
Internal/external	59/36
Macroscopic hematuria	15
Loss of weight	5
Malignity	20

Macroscopic hematuria was seen in 15 patients. Of these, five were diagnosed with ureter tumors, and four were diagnosed with urinary tuberculosis. The frequency of malignancy in patients with hematuria (5/15) was two times greater than in patients without hematuria (15/85; 33% vs. 17.6%; p = 0.01). Nephroureterectomy (NPU) was performed in all patients with ureteral tumors and in two patients with tuberculosis. The cases with hematuria needed significantly higher nephrectomy (7/15; 46.6%) than those without hematuria (10/85; 11.7%; OR: 4; p = 0.001).

Significant weight loss was observed in five patients. Of these patients, cervical metastasis was found in three, gastric cancer in one, and lymphoma in one. The malignancy rate was significantly higher in patients with weight loss (5/5; 100%) than in those without weight loss (15/95; 16%; OR: 6.25; p < 0.001).

Upper urinary tract etiology

The cause of HN in 50 patients was due to upper urinary tract system pathologies. A primary ureteropelvic junction (UPJ) stenosis was found in 10 patients. CTU was not convenient for the lumen examination because the contrast material did not pass through the ureter. Following that, a magnetic resonance urography (MRU) was performed in patients. Patients with suspected UPJ stenosis were examined with ureteroscopy (URS) and retrograde ureteropyelography (RPG due to their age over 20 years. Ureterovesical junction (UVJ) stenosis was detected in 13 patients. Congenital UVJ stenosis was considered in these patients who had no previous surgery or stones. In three of these 13 patients, the stenosis was caused by ureteroceles. Diuretic renal scintigraphy was performed to determine the obstruction of definite primary UPJ or UVJ stenosis.

Obstructive findings were not fully demonstrated in any of the patients; they were accepted as mild stenosis, with the nuclear material clearing late. Stenosis was detected in eight patients in the different levels of the ureter. In four of these eight patients, a kink of the middle ureter, two high ureteral insertions to the pelvis, and primary stenosis of the middle ureter in two patients were determined. The diagnoses were confirmed in all patients using diagnostic URS and RPG. The ureter stones were detected during diagnostic URS in two patients who did not show any stone on CT. The stone of both patients was observed to be of 4 mm and embedded in the ureter wall. A total of seven patients had ureteral tumors, five of whom had at least one macroscopic hematuria attack, while two of them did not. In our three patients who underwent URS for ureteral stone at another center one year ago, we detected stenosis. Retrocaval ureter diagnosis was made by RPG in two patients who could not be diagnosed despite CTU results. Voiding cystourethrography (VCUG) was performed, and bilateral reflux was diagnosed on the third of the dilated ureter. The Yo-Yo reflux phenomenon was considered in two patients due to the presence of a duplicated ureter in CTU and non-pathological diagnostic URS. Dynamic renal scintigraphy demonstrated enhancement of the lower pole of the kidney, thus confirming the Yo-Yo reflux phenomenon. URS was performed in a patient who had a filling defect in the upper part of the ureter on MRU, and biopsy showed a fibroepithelial polyp.

Lower urinary tract etiology

The cause of HN in 11 patients was due to the lower urinary system. In 10 of them, the problem was in the bladder; the urethral stenosis was another cause. Uroflowmetry was not performed because our patients with urethral stenosis did not have LUTSs. We diagnosed a type 3 posterior urethral valve with urethrocystoscopy. We detected neurogenic bladder in seven patients with bladder origin. Among these patients, four of them had diabetes, two had previous spinal surgery, and one had neurosyphilis. All patients with a diagnosis of the neurogenic bladder had complaints of urinary incontinence and frequent urination, and the diagnosis was confirmed by cystometry. A bladder tumor was detected in two patients. Muscle invasion has been reported in patients without previous positive cystoscopy findings who have undergone random transurethral resection. These patients underwent radical cystectomy, and their results reported extravesical extension. We identified a grade 4 cystocele as the cause of bilateral HN during diagnostic urethroscopy and vaginal examination in another patient.

Other pathologies

HN caused by extrinsic compression into the ureter was detected in a total of 23 patients. There was a pressure resulting from the accessory vessel in four of them: three originated from the aorta and one from the common iliac artery. HN due to urine collecting system compression was detected in 11 patients. Metastatic lymphadenopathy was the etiological factor in eight of these cases, metastasis of the stomach cancer into the recto-vesical space in one case, and direct invasion of the mass in two cases. One of the two patients who developed HN by malign invasion was diagnosed with colon cancer, and the other with endometrium cancer. In four patients who developed HN due to iatrogenic causes, we observed that according to the exploration results, the ureter was sutured or sealed with a ligature; gynecological surgery was performed in two of them, gastroenterological surgery in one, and urological surgery in one. None of the patients knew about an injury in their ureter in the postoperative period, and probably injuries were not noticed during the operation. Diagnostic biopsy was not performed following immunosuppressive therapy. Curative treatment was confirmed by the disappearing HN, without the need for a double-J stent or nephrostomy.

The number of patients with urinary tuberculosis was 10, with unilateral HN due to kidney and ureter involvement in 4 patients and bilateral HN due to bladder involvement in 6 patients. In a patient with uncontrolled diabetes, we detected the cause of HN as bladder and ureteral candidiasis (Table 2).

**Table 2:** Causes of hydronephrosis in our patients UVJ, ureterovesical junction; URS, ureteroscopy; LAP, lymphadenopathy

Causes		Number (%)	
Upper urinary tract		50 (50)	
Ureteropelvic junction stenosis		10 (10)	
			Ureterovesical junction stenosis	Primary UVJ stenosis	10 (10)	
				Ureterocele	3 (3)	
Primary ureteral stenosis		2 (2)	
Ureteral kink		4 (4)	
High insertion of the ureter to the pelvis		2 (2)	
Ureteral stone		2 (2)	
Ureteral tumor		7 (7)	
Stricture after URS		3 (3)	
Retrocaval ureter		2 (2)	
Vesicoureteral reflux		3 (3)	
Yo-Yo reflux phenomena		2 (2)	
Ureteral polyp		1 (1)	
Lower urinary tract		11 (%11)	
Neurogenic bladder		7 (%7)	
Bladder tumor		2 (%2)	
Urethral stricture		1 (%1)	
Cystocele		1 (%1)	
External		23 (23)	
Aberrant renal artery		4 (4)	
Retroperitoneal mass	Metastatic LAP	8 (8)	
	Metastatic tumour	1 (1)	
	Invasion	2 (2)	
Iatrogenic	Gynecology	2 (2)	
	General surgery	1 (1)	
	Urology	1 (1)	
Retroperitoneal fibrosis		4 (4)	
Others		11 (11)	
Tuberculosis	Kidney-ureter	4 (4)	
	Kidney-ureter-bladder	6 (6)	
Urinary candida		1 (1)	
Non-refluxive and non-obstructive		5 (5)	
Total		100 (100)	

## Discussion

In our opinion, it would be beneficial to review the test results of patients with HN to determine the etiology of the condition. US is the primary imaging method used for almost every subject in urology. Because US is inexpensive, harmless, and easily accessible, it can be used in the diagnosis and follow-up of HN [3]. It should be remembered, however, that there is up to 35% false negativity in the acute phase [4]. Since our patients had previously undergone US, it was also used in the follow-up. To increase the sensitivity of the US, Doppler US (CDUS) was considered to differentiate between actual obstructions. CDUS was reported to have a cut-off value of 0.7 on the resistivity index (RI) [5]; however, subsequent studies have shown that the RI is neither specific nor sensitive [6]. US is being increasingly recognized for its ability to differentiate between the ureter, crossing vessel, and UPJ obstruction, and it is even being considered as a candidate diagnostic method alone. The sensitivity of CDUS to identify the crossing vessel is 90% and that of contrast-enhanced CDUS is 100% [7-8]. No color Doppler or contrast Doppler examination was used as a primary diagnostic tool in any of the four patients in our series. Two of our patients were diagnosed using high-resolution CT angiography. The balance of the cases was found during a diagnostic laparoscopy.

Another cause of HN, where US alone is the diagnostic tool, is bladder cancer. The sensitivity of US is 96% in bladder cancer and around 87% in non-muscle invasive cancer [9-10]. Primary carcinoma in situ (CIS) accounts for only 3% of all bladder tumors. However, it is challenging to detect primary CIS cases using imaging methods, and therefore it can only be diagnosed by bladder biopsy [11]. In our series, neither US nor CT was capable of detecting bladder tumors in two patients who were later diagnosed through bladder biopsies.

Excretory urography (EU) is not preferred as a primary tool due to the sensitivity of the CT in detecting ureteral stones and because it provides information about the etiology of non-urinary causes of obstruction [12]. However, in cases with obstruction, the ureteral passage of the contrast agent may be delayed and may not be visible on CTU. Multiple re-shoots may help monitor the contrast agent even if it is passed too late during EU. Particularly in iatrogenic injuries of the ureter, the location and length of the injured part can be determined using this method [13]. In our study, among four patients with iatrogenic injury, EU was used as the primary diagnostic tool and combined with RPG to manage the cases. RPG can be used in cases in which imaging cannot be achieved using other contrast-enhanced methods or in case the patient cannot be given a contrast agent. In our series, the diagnosis was made by RPG in two patients with retrocaval ureters, four with ureteral kinks, and two patients with high pelvic insertion of the ureter.

The primary imaging method in patients with residual HN is non-contrast CT (NCCT). NCCT is superior to EU in terms of detecting decreasing parenchymal density, perinephric linearization, increases in kidney size, ureteric dilatation levels, and ureteral stones [14]. None of the patients in our series could be diagnosed with US or NCCT. NCCT has 96%-100% sensitivity in identifying ureter stones. However, if the ureter stones are smaller than the cross-sectional area, the stone may not be seen [15]. CT slices usually provide 5-mm cross-sections. Stones that are less than 5 mm and cause less HN than larger ones therefore cannot be detected [16]. Ureteral stones could not be detected using CT in two of our patients but were diagnosed during URS. If NCCT cannot provide a diagnosis, contrast-enhanced CT should be performed. In some patients, when CTU is combined with EU, it can offer excellent diagnostic value. Notably, for UPJ stenosis and/or ureteral lesions, CTU can provide the most valuable data [17]. Similarly, MRU can be as effective as CTU. Both these methods are distinctive because of the detailed retroperitoneal imaging they provide [18]. In our series, 9 of 11 patients with external ureteral compression were diagnosed using CTU and 2 using MRU, and definitive diagnoses were established later through biopsy. For the four patients who were diagnosed with retroperitoneal fibrosis, contrast-enhanced MRI was used. The preliminary diagnoses were made using MRU in 20 patients with primary UPJ and UVJ stenosis. Later, diagnostic URS confirmed these diagnoses. MRU offers more detailed data with a two-phasic technique, and image quality can be further enhanced with hydration and diuretics [19]. MRU can therefore provide more detailed images of the ureter and pelvis. This makes it possible to distinguish between primary UPJ or UVJ stenosis causes, external pressure, ureter tumors, and ureteral benign polyps [20-21]. The preliminary diagnoses of seven patients with ureter tumors and one patient with a fibroepithelial polyp were made using MRU and confirmed by diagnostic URS and biopsy.

VCUG is the gold standard for a vesicoureteral reflux (VUR) diagnosis [22]. VUR was diagnosed in three patients with ureteral dilation as a result of diagnostic URS by VCUG. A renal scan is an effective method for detecting true obstruction in the presence of HN [23]. However, it plays no role in determining their etiology. In our series, dynamic renal scintigraphy was used to determine whether HN was obstructive in patients with primary UPJ and UVJ stenosis. Urodynamic examination is the cornerstone in the diagnosis and management of neurogenic bladder [22]. Urodynamic examinations were performed in seven patients with irritative LUTS in our study. As a result, a low-compliant and low-capacity bladder was diagnosed in each case. In the Yo-Yo reflux phenomenon, the main diagnosis is based on dynamic renal scintigraphy [24]. Two patients in our series were diagnosed with the Yo-Yo reflux phenomena by dynamic renal scintigraphy.

The urinary system is the third most common extrapulmonary location of tuberculosis after the pleura and lymph nodes and is affected in 10% of all tuberculosis patients [25]. The kidney is the most affected organ in the urogenital system [26]. Ureter involvement is directly from the kidney. It is most commonly located in the distal ureter and causes UVJ stenosis. The most common placement are the entrance of ureter to bladder and trigone in the bladder. The result of fibrosis is a low-compliant, low-capacity bladder. However, in patients with urinary tuberculosis, HN is most commonly caused by UVJ stenosis even if the bladder is involved [27]. In our series, our patients had HN and ureter involvement. Four of our patients had unilateral HN, and the other six patients had bilateral HN. The diagnosis is usually made on the first urine of the day with a tuberculosis survey and tuberculosis culture. However, in recent years, polymerase chain reaction (PCR) has become popular with high specificity and sensitivity [28]. Although we repeat the tuberculosis study at least three times, only 6 of 10 patients had a positive result. In the others, the diagnosis was made by the PCR test.

Candida Albicans associated HN is very rare. It is usually seen in patients who have a problem with the immune system [29]. The patient in our series had diabetes mellitus for 20 years, and HN developed due to the accumulation of candida balls. The vast majority of cancer patients have significant weight loss, and 20% of cancer patients lose their lives due to weight loss [30]. In our study, we found malignancy in all of our patients with weight loss. Weight loss had a 6.25 times and hematuria two times greater chance of malignancy in those without HN.

## Conclusions

Any patient who is admitted due to HN may not be diagnosed with the US and CT.. The laterality of HN and obstruction level should be determined. CTU or MRU are indispensable imaging methods. However, diagnostic invasive endoscopic procedures may also be required. Macroscopic hematuria and weight loss are poor prognostic parameters but are often seen in life-threatening diseases such as malignancy.
